# Reproductive Strategies and Population Genetic Structure in Two Dryland River Floodplain Plants, *Marsilea drummondii* and *Eleocharis acuta*

**DOI:** 10.3390/genes13091506

**Published:** 2022-08-23

**Authors:** William Higgisson, Linda Broadhurst, Foyez Shams, Bernd Gruber, Fiona Dyer

**Affiliations:** 1 Centre for Applied Water Science, University of Canberra, University Drive, Bruce, Canberra, ACT 2617, Australia; 2 Centre for Australian National Biodiversity Research, CSIRO National Research Collections Australia, GPO Box 1700, Canberra, ACT 2601, Australia; 3 Centre for Conservation Ecology and Genetics, University of Canberra, University Drive, Bruce, Canberra, ACT 2617, Australia

**Keywords:** propagule biology, aquatic plants, semi-arid and arid, floodplain, dispersal, reproduction

## Abstract

Aquatic plants share a range of convergent reproductive strategies, such as the ability to reproduce both sexually and asexually through vegetative growth. In dryland river systems, floodplain inundation is infrequent and irregular, and wetlands consist of discrete and unstable habitat patches. In these systems, life history strategies such as long-distance dispersal, seed longevity, self-fertilisation, and reproduction from vegetative propagules are important strategies that allow plants to persist. Using two aquatic plants, *Marsilea drummondii* and *Eleocharis acuta*, we investigated the proportions of sexual and asexual reproduction and self-fertilisation by employing next-generation sequencing approaches, and we used this information to understand the population genetic structure of a large inland floodplain in western New South Wales (NSW), Australia. Asexual vegetative reproduction and self-fertilisation were more common in *M. drummondii*, but both species used sexual reproduction as the main mode of reproduction. This resulted in a highly differentiated genetic structure between wetlands and a similar genetic structure within wetlands. The similarity in genetic structure was influenced by the wetland in the two species, highlighting the influence of the floodplain landscape and hydrology on structuring population genetic structure. The high levels of genetic variation among wetlands and the low variation within wetlands suggests that dispersal and pollination occur within close proximity and that gene flow is restricted. This suggests a reliance on locally sourced (persistent) seed, rather than asexual (clonal) reproduction or recolonisation via dispersal, for the population maintenance of plants in dryland rivers. This highlights the importance of floodplain inundation to promote seed germination, establishment, and reproduction in dryland regions.

## 1. Introduction

Aquatic plants are an extremely heterogeneous group of species sharing a range of convergent reproductive and other life history traits, which allow them to live and thrive in aquatic environments [1,2]. One of the distinguishing reproductive strategies shared by most aquatic plants is asexual reproduction, which includes the production of seed without fertilisation (agamospermy) and vegetative reproduction [1]. Although vegetative reproduction is common in aquatic plants, most species retain the ability for both sexual and asexual reproduction (e.g., *Phragmites australis* [3], *Decodon verticillatus* [4], and *Cladium jamaicense* [5]). The challenges of successful fertilisation in aquatic environments have also led to the evolution of some unique strategies, such as hydrophily, where pollen is transported under water [6], and pollination and seed set within closed flowers (e.g., in *Callitriche* [1]).

Sexual reproduction increases genetic diversity, while asexual reproduction maintains the genetic uniformity of well-adapted gene complexes [6,7]. The higher genetic variability in sexual propagators facilitates adaption for long-term persistence, whereas asexual propagators promote short-term adaption, which can be beneficial for rapid establishment and expansion [2]. Li [2] proposed that asexual reproduction in aquatic plants is a strategy that ensures population maintenance, while sexual reproduction is responsible for population restoration from extreme events on evolutionary timescales. If this is the case, we may expect that aquatic plants will have highly differentiated populations with little among-population gene flow and extensive within-population clonality.

Asexual reproduction provides an alternative reproductive strategy when sexual recruitment is challenging or limited. Barrett [8] observed that in *Eichhornia crassipes* (water hyacinth), sub-optimal seed production was related to inefficient pollinator service and seedlings were only recorded at sites with saturated soil and shallow water conditions. In the aquatic macrophyte *Phragmites australis* (common reed), seed production was found to be highly variable between sites and years, and was related to rainfall and temperature [9]. Interspecific variation in sexual and asexual reproduction was observed in the aquatic plant *Decodon verticillatus* (swamp loosestrife), where asexual reproduction became more dominant toward the geographical range limits of the species. This was caused by reduced sexual performance related to genetic traits (such as reduced pollen tube growth and ovule penetration) and ecological factors (primarily changes in temperature) [4]. For this species, the traits involved in sexual reproduction in the northern population contributed little to fitness and, as such, are likely to have been disabled through genetic mutations [10].

In vegetative (asexual) reproduction, there are two levels of population structure that can provide meaningful data on plant demography: the ‘ramet’, which is the unit of clonal growth (i.e., a single individual plant produced by clonal propagation), and the ‘genet’, which is the colony of all the genetically indistinct ramets (i.e., there can be many ramets within a genet) [11]. The organs of clonal propagation (for example, tillers, rhizomes, or bulbs) influence the spatial arrangement and size of ramets and the amount of outcrossing that may occur between ramets [12]. The size of a clone has been found to influence sexual reproduction, with larger clones found to be increasingly saturated with pollen arriving from the same clone in *Carex platyphylla* (broad-leaf sedge) [13].

A range of factors are likely to influence the population genetic structure in aquatic plants, such as hydrology [14] and the river and floodplain topography, as well as plant morphology, pollination systems [15], the modes and rates of extinction, dispersion and colonisation [16], and sexual and asexual propagule dispersal [17] and establishment [18]. While rivers facilitate the movement of individuals and genes [14], high levels of genetic differentiation among populations and low within-population variation is often observed in aquatic plants, and may be related to widespread asexual (clonal) reproduction [19]. Significant genetic differentiation between populations was observed in *Cladium jamaicense* (swamp sawgrass), with the genetic structure reflecting patterns from colonisation maintained through long-lived clones [5]. Similarly, Piquot et al. [20] observed genetically discrete populations consisting of a single or few clones in *Sparganium erectum* (branching bur-reed).

One reason for the high genetic differentiation often observed among populations is that long-distance dispersal (>100 m) is rare in plants [21,22]. Despite this rarity, long-distance dispersal does occur and is essential for colonising new suitable habitats [22]. It is of particular importance for unstable local populations, which may be more prone to localised extinction events [23]. The flow regime of rivers in dryland regions is highly variable, where floodplain inundation is infrequent, irregular, and reliant on rainfall during particularly wet years [24]. This means that floodplain wetlands may be discrete and possibly unstable habitat patches [23], with aquatic plants in these habitat patches naturally going through localised extinction and colonisation events. Here, long-term population survival relies on long-distance dispersal [22,25] and the ability of seeds to remain dormant and viable between flooding events. If floodplain habitats in dryland regions are metapopulations, the extinction rates and modes (and rates) of recolonisation (either from the entire metapopulation (migrant pool) or from a single-source population (propagule pool)) will influence population genetic structure [16,26]. For example, repeated extinction and population turn-over in metapopulations reduces the within-population diversity and increases the among-population diversity, especially in the absence of migration between extant populations [26].

The genetic distinctiveness of individuals has long been a challenge in plant demography, especially for species that are clonal, where a plant census is unsuitable [11]. Recent advances in genomics have made the identification of ramets and their genets more achievable, allowing the extent of clonality in aquatic plants such as *Laminaria rodriguezii* (kelp) [27] and *Chara* spp. [28] to be determined. Understanding the population genetic structure of aquatic plants provides information, including the rate of sexual and asexual reproduction, to guide and predict the consequences of land or water management actions on these species.

The Murray–Darling Basin covers an area of 1 million km^2^ in the southeast of Australia and is an important region of Australia for agricultural production as well as supporting around 2.3 million people and a range of environmental assets [29]. Many rivers that make up the Murray–Darling Basin occur in semi-arid and arid regions and are characterised by low and extreme variability in flows [24]. These systems have undergone pronounced changes over the last century through river regulation [30,31]. 

Two aquatic/semi-aquatic plants that reproduce sexually and asexually via vegetative growth and are common on the floodplains of the Murray–Darling Basin are *Marsilea drummondii* A. Braun (common nardoo) and *Eleocharis acuta* R.Br. (common spike-rush). On the floodplains of the Murray–Darling Basin, *M. drummondii* and *E. acuta* are often the dominant groundcover during and after flooding events, forming monospecific stands or clumps (Pers Obs. W Higgisson and F Dyer). Being common and widely distributed, *M. drummondii* and *E. acuta* play an important role in maintaining functioning river floodplain ecosystems within the Murray–Darling Basin. Using these two species, we investigated the extent and spatial arrangement of (1) asexual vegetative propagules, (2) self-fertilisation, and (3) plants in a parent–offspring relationship across a large inland floodplain in western New South Wales (NSW), Australia using next-generation sequencing approaches. Using this information, we also explored the population genetic structure of both species. Understanding the population genetic structure of these plants provides important information on mating patterns, resource allocation, and the role of hydrology in the maintenance and establishment of these and other plants on floodplains in dryland regions.

## 2. Materials and Methods

### 2.1. Study Area

**Figure 1:**
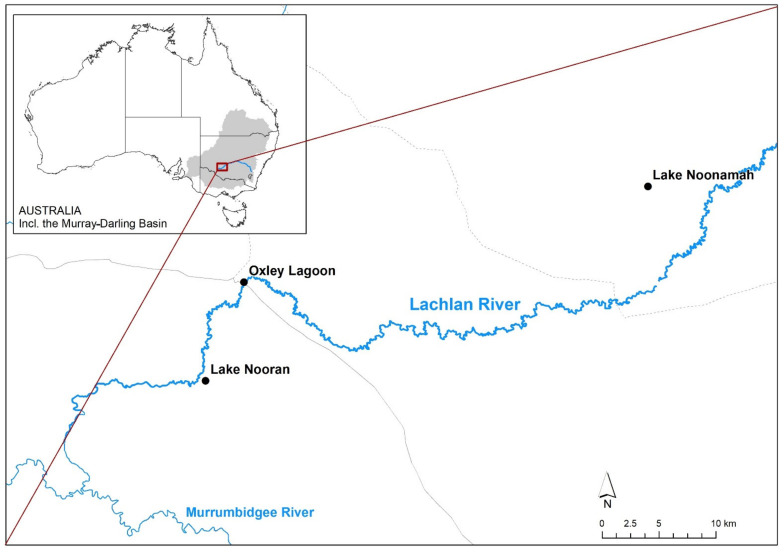
The floodplain wetland sites used in this study, where *Eleocharis acuta* and *Marsilea drummondii* samples were collected from within 2–3 patches in each wetland. The inset shows the location of the Lachlan River (in blue) and the Murray–Darling Basin (shaded light grey) in Australia.

This study was carried out in three wetlands on the floodplain of the lower Lachlan River, NSW, Australia (Figure 1), where both species occur. The lower Lachlan River has a semi-arid climate that experiences a mean annual rainfall of 319 mm at Oxley, NSW (station number: 049055) [32]. The temperature ranges from a mean monthly summer maximum of 35.1 °C in January to a mean monthly winter maximum of 15.8 °C in July (at Hay (Airport), NSW, (station number: 075019) [33]). The lower Lachlan River is characterised by numerous distributary channels and anabranches, and vast areas of floodplain wetlands [31]. The highly variable flow regime of the Lachlan River means that wetland inundation is irregular and infrequent [30].

### 2.2. Study Species

*Marsilea drummondii*

*Marsilea drummondii* (*Marsileaceae*) is an aquatic/semi-aquatic perennial fern widely distributed across inland, mainland Australia [34]. It is a submerged plant with leaves floating on the water surface and occurs at the edge of lakes, swamps, and floodplains. The fronds are clover-like, growing at the end of stalks which vary in length from 2 to 30 cm.

*Marsileaceae* is an aquatic and semi-aquatic heterosporous perennial rooted water fern that forms rhizomatous spreading clumps [35]. Ferns such as this alternate between haploid and diploid phases, with the diploid phase being the most visible. *Marsilea* produces a modified seed-like spore-bearing leaf known as a sporocarp, which has a thick sclerenchymatous wall that protects the enclosed sori against damage, such as insect attacks and dryness [36]. When stimulated by water, the sporocarp produces sporangia, which give rise to haploid male microspores and female megaspores, and with internal fertilisation giving rise to the next diploid generation. *Marsilea drummondii* sporocarps are 4–9 mm long, on stalks 8–35 mm long [37].

Reproduction in *Marsilea* is highly dependent on the flooding regime and the wetting and drying cycles, with the entire reproductive biology dependent on water for the release of spores from the sporocarp, the dispersal of spores following fertilisation, and the sinking of young developing embryos [38]. Sporocarp fruiting in *Marsilea* (including in *M. drummondii*) occurs primarily on mud as a habitat dries [39]. Viable sporocarps of several *Marsilea* species have been reported to germinate from herbarium material up to 100 years old [40], highlighting the ability of this genera to remain viable for long periods. *Marsilea drummondii* has germinated in soil seed bank studies, indicating their ability to remain viable and contribute to the soil seed bank (Higgisson et al. 2021, unpublished).

The sporocarp can be transported by adhesion, such as to the external surfaces of birds, and can be ingested [39]. The sporocarps of *M. mucronata* have been shown to pass intact through the digestive tract of waterbirds [41]. The size and weight of sporocarps (sporocarps in *M. drummondii* range from 4 to 9 mm long) are predicted to limit wind dispersal [35]. Hydrochory is likely to be an important dispersal mechanism for sporocarps during flooding events; however, sporocarps start to open within a few hours of contact with water [38]. Once the sporocarp opens, the spores are short-lived and must germinate quickly. They are highly susceptible to drying; therefore, spores are unlikely to be units of long-distance dispersal and dormancy [38].

*Eleocharis acuta*

*Eleocharis acuta* (*Cyperaceae*) is a rhizomatous perennial plant that grows to 90 cm high [37,42]. *Eleocharis acuta* occurs along the eastern and southwestern mainland of Australia, and across Tasmania as well as New Zealand, New Guinea, and Norfolk Island [42]. It grows in moist conditions along streams, roadsides, and floodplains and around dams.

*Eleocharis acuta* seeds can float on water (50% of seeds floated for >30 days), and the species is likely to disperse via hydrochory; the seeds are dormant, although the mechanism involved is unknown, and they require extended contact with water to germinate (>40 days) [43]. Intact *E. acuta* seeds were recorded in waterbird faecal samples by Green et al. [44], suggesting that the internal transport of seeds by waterbirds may be an important dispersal mechanism. All members of the *Cyperaceae* are wind-pollinated [45]. While the timing of spikelet maturity is unknown for *E. acuta*, the spikelet of *E. laeviglumis* from southern Brazil is dichogamous and protogynous, with its reproductive organs maturing at different times [46], which reduces self-pollination and promotes out-crossing.

### 2.3. Study Design and Sampling Strategy

**Figure 2:**
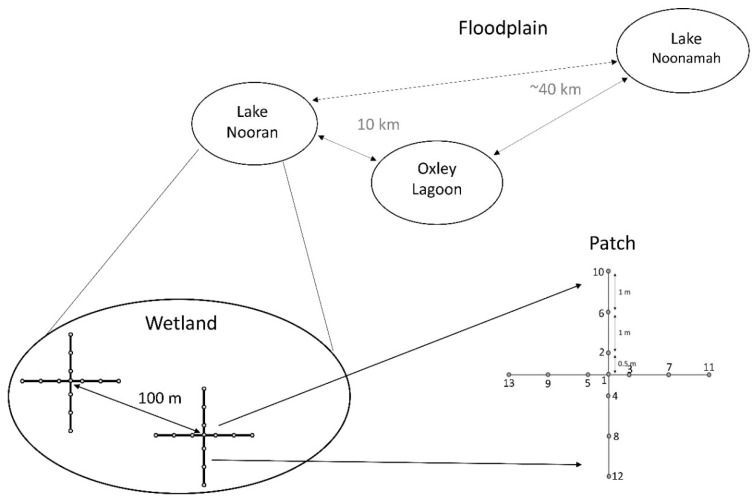
Conceptual diagram showing the three wetlands used in this study, how patches were arranged within each wetland, and how samples were collected within each patch.

Leaf samples of *M. drummondii* and *E. acuta* were collected from Lake Nooran, Oxley Lagoon, and Lake Noonamah. Lake Nooran is an open temporary wetland a few hundred metres from the main channel of the Lachlan River, in a complex of wetlands where the Lachlan River terminates. Oxley Lagoon is in close proximity (<50 m) to the Lachlan River up-stream of Lake Nooran, while Lake Noonamah is on an ephemeral channel off the main stem of the Lachlan River (Figure 1). Within each wetland, a total of 13 leaf samples were collected from within 2 or 3 patches (Figure 2) that were approximately 100 m apart. Sampling at each patch consisted of 13 points, starting from a central point at which a single leaf was collected. From the central point, the nearest leaf was collected from points 0.5 m north, east, south, and west, then 1.5 m from the central point, and finally 2.5 m from the central point in the same pattern (Figure 2). A total of two patches were sampled within Lake Nooran and Oxley Lagoon and three patches at Lake Noonamah for each species. This resulted in a total of 91 samples for each species.

### 2.4. Genotyping

DNA extraction was performed using protocols developed by Diversity Array Technology Pty Ltd. (DArTseq™, Canberra, Australia). Tissue samples (25 mg) were incubated overnight at 56 °C with lysis buffer and proteinase K. Lysed tissue was washed using a washing buffer to remove impurities, such as proteins and polysaccharides, and stored in an elution buffer. Genotyping was undertaken on samples from each species with a genome-wide profiling approach using restriction enzymes to produce DNA fragments, which were pooled (for each species) and sequenced using next-generation platforms by Diversity Arrays Technology, Canberra, Australia (http://www.diversityarrays.com accessed 1 July 2021). DArTseq™ represents a combination of a DArT complexity reduction method and next-generation sequencing platforms [47,48]. Therefore, DArTseq™ represents an implementation of sequencing complexity-reduced representations [49] and more recent applications of this concept on the next-generation sequencing platforms [50,51]. A detailed description of the DArTseq™ methodology is given by Killian et al. [48]. DarTseq genotyping produced 45,940 single nucleotide polymorphisms (SNPs) for *M. drummondii* and 150,801 SNPs for *E. acuta*.

### 2.5. Data Analysis

The SNP data supplied by DarTseq and associated metadata were read and filtered using the software package dartR, Version 1.9.1 [52]. As this research was interested in determining the relatedness of samples and their spatial arrangement within and among wetlands, we initially investigated pairwise frequencies in consistent loci. We determined three levels of pairwise relatedness being (1) genetically identical pairs through asexual clonal reproduction; (2) pairs in a parent–offspring relationship via self-fertilisation; and (3) pairs in a parent–offspring relationship via out-crossing (Table 1). Under these three pairwise relatedness scenarios, the true number of inconsistent loci in each scenario should be zero. Since it is possible that PCR error might result in small differences between identical samples, we filtered the data based on a read depth ≤12 and ≥50 for *M. drummondii* and a read depth ≤15 and ≥50 for *E. acuta* (considering the much larger starting dataset), which removed or significantly reduced the occurrence of SNP errors in the datasets. We also filtered the data for average repeatability of alleles at a locus of 99.8% and a call rate threshold of 0.95; all secondary SNPs where then removed. This filtering left 1241 SNPs for *M. drummondii* and 6618 SNPs for *E. acuta*, which were used to determine the three levels of pairwise relatedness. 

We have provided the results from the relatedness analysis for each species as S1 for *E. acuta* and S2 for *M. drummondii*, showing the number of inconsistent loci and the total number of loci used in the analysis between each pair of samples in each of the three relatedness scenarios. We reported only the pairs with no (0) locus differences in the results, but highlighted that those pairs with a single or few locus differences may also be in the relatedness scenarios.

Following the relatedness analysis, a single representative of each genotype (i.e., the genetically identical pairs determined in Scenario 1) from each patch was retained (i.e., one of each clonal group per patch), leaving 84 *M. drummondii* and 90 *E. acuta* genotypes for further analysis. These datasets were then re-filtered as described above to produce a dataset of 1242 SNPs for *M. drummondii* and 6623 SNPs for *E. acuta*.

The observed (*H*_o_) and expected (*H*_e_) heterozygosity were calculated for each species across all samples, within each wetland and within each patch using the function gl.report.heterozygosity from the package dartR [52]. An inbreeding coefficient (*F*_IS_) was calculated for each patch and for each species, using the function boot.ppfis in the package Hierfstat, version 0.5.7 [53], which uses the formula *F*_IS_ = 1−(*H*_o_/*H*_e_), with 1000 bootstraps.

Principle coordinates analysis (PCoA) was undertaken on all genotypes for each species in dartR, version 1.9.1 [52]. The genetic structure was estimated using the program Structure, version 2.3.4 [54]. The number of populations tested was assumed to be ‘K’ = from 1 to 10. Using an admixture model, the analysis was run 10 times for each ‘K’ value, with burnin length and MCMC replication after burnin. The optimal values of ‘K’ (number of panmictic clusters) were identified for each species by following Evanno′s method [55] using Structure Harvester [56]. The overall genetic differentiation was estimated using Analysis of Molecular Variance (AMOVA), undertaken using the function poppr.amova in the package poppr, version 2.9.2 [57], using the ade4 implementation of AMOVA. The level of genetic differentiation among patches and among wetlands was assessed by calculating the *F*_ST_ values using the package GenAlEx, version 6.5 [58].

## 3. Results

### 3.1. Clonality

The 91 samples for each species collected from two patches in Lake Nooran and Oxley Lagoon and three patches in Lake Noonamah (13 samples per patch) yielded 83 genotypes for *M. drummondii* and 90 for *E. acuta* (Table 2). Seven *M. drummondii* samples from within Lake Nooran’s Patch 1 were genetically identical, with samples distributed over an area of approximately 2 m^2^ (see Figure S1 for the spatial arrangement of samples). There were also three genetically identical *M. drummondii* samples from Lake Noonamah: two from Patch 1 (Samples 1 and 12, which were 2.5 metres apart) and the third from Patch 2 (Figure S1). In *E. acuta*, there was one pair of samples that were genetically identical in Lake Noonamah’s Patch 2 (50 cm apart). No other patches contained clones for either species.

### 3.2. Parent–Offspring Relationship through Self-Fertilisation

For *M. drummondii*, we only detected pairwise samples in a parent–offspring relationship via self-fertilisation in Lake Noonamah (Table S1), where one sample in Patch 1 was related to three other Patch 1 samples, with a 4 m distance between these samples (Figure S1). A sample in Lake Noonamah’s Patch 2 was observed to be in a parent–offspring relationship via self-fertilisation with two samples from Patches 1 and 2. There were three Lake Noonamah Patch 3 samples in a parent–offspring relationship via self-fertilisation, with a further four samples from the other two Lake Noonamah patches (three samples in one patch and one in the other) that were also in a parent–offspring relationship via self-fertilisation. In *E. acuta*, there was only one pair of samples in a parent–offspring relationship via self-fertilisation, which occurred in Lake Noonamah (100 cm apart) (Figure S2).

### 3.3. Parent–Offspring Relationship through Outcrossing

In *M. drummondii*, a total of 422 (or 10% of the total) pairs were in a parent–offspring relationship through outcrossing (Table S1). The majority of these pairs occurred within and between patches within a wetland, with slightly more than half (52%) of these occurring between samples within a patch, and ~47% occurring between samples in different patches within the same wetland. Three samples in Oxley Lagoon’s Patch 2 were in a parent–offspring relationship through outcrossing with three samples in Lake Nooran. In *E. acuta*, only 34 pairs of samples were in a parent–offspring relationship through outcrossing. These pairs only occurred within patches, and not between patches or wetlands.

### 3.4. Other Sample Pairs with Few Locus Differences

The results from pairwise relatedness analyses based on the three relatedness scenarios for *M. drummondii* (Table S1) and *E. acuta* (Table S2) also highlighted that there were some genotype pairs in both species with only a single or few locus differences in the three relatedness scenarios. In *M. drummondii*, there were 12 pairs that had a single locus difference in the clonal relatedness scenario and a further 28 pairs that had a single locus difference in the parent–offspring by self-fertilisation scenario (of 1238 loci). In *E. acuta*, there were three pairs that had between two and five inconsistent loci (out of 6588 loci) in the clonality scenario, while the next most similar pair had 41 locus differences (see Table S2). These pairs could be genetically identical through clonal reproduction or in a parent–offspring relationship through self-fertilisation, given the potential for sequencing errors.

### 3.5. Genetic Diversity

The observed and expected heterozygosity varied between the two species. *M. drummondii* had a mean observed heterozygosity (*H_o_*) across all genotypes of 0.105 compared with *E. acuta*, which had a mean *H_o_* of 0.165 (Table 3). Despite this difference, similar patterns for *H_o_* and *H_e_* were observed between the two species across the different wetlands and patches. For example, *H_o_* and *H_e_* were lowest for both species at Lake Noonamah, compared to the same measures at Lake Nooran and Oxley Lagoon. Further, at the patch level, *H_o_* and *H_e_* were the lowest at Lake Noonamah’s Patch 3 compared with the other patches. *H_e_* was the greatest for both species at Oxley Lagoon, and within Oxley Lagoon’s Patch 1 at the patch level. The highest *H_o_* was observed at Oxley Lagoon in *M. drummondii* and Lake Nooran in *E. acuta* (Table 3). The inbreeding coefficient (*F*_IS_) for *M. drummondii* was greater than 0 at Lake Noonamah’s Patches 1 and 2, and slightly greater than 0 at Oxley Lagoon’s Patch 2, which indicated that inbreeding had occurred at these patches. In *E. acuta*, *F*_IS_ was less than 0 in all patches, demonstrating excess heterozygosity as compared to expected in all patches, and the widespread occurrence of outcrossing (Table 3).

### 3.6. Population Genetic Structure

**Figure 3:**
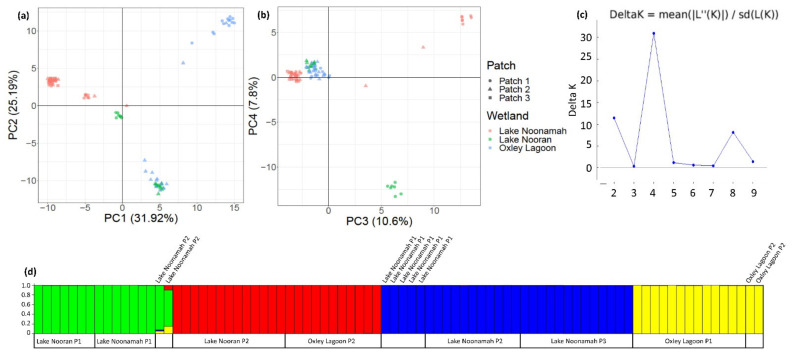
Plots showing (**a**) axes 1 and 2 and (**b**) axes 3 and 4 from PCoA analysis of 84 *Marsilea drummondii* genotypes across three wetlands (2–3 patches per wetland) in the lower Lachlan River Catchment; (**c**) representation of the Akaike value used to determine the number of populations (K); and (**d**) representation of the probability of population assignment for each individual genotype, coloured by cluster and ordered by wetland and patch.

The first two PCoA axes accounted for 57% of the total variation in *M. drummondii*, with similar variation along both axes (31.9% vs. 25.2%). Most samples were clustered within the wetland patch from which these were collected (Figure 3a), with several discrete clusters present. At the wetland level, the Lake Noonamah plants were differentiated from all others and fell into two clusters, apart from one sample that fell near a small group of Lake Nooran plants. The remaining Lake Nooran plants grouped with some of the Oxley Lagoon plants, with the remainder of clustering some distance away. At the patch level, most plants were grouped together, with some exceptions such as an Oxley Lagoon Patch 2 plant falling close to the Patch 1 samples at this site and those at Lake Noonamah. The third and fourth PCoA axes showed further clustering and highlighted the further divergence of some patches. The third axis accounted for a further 10.6% of the total genetic variation and highlighted a strong differentiation of samples collected in Lake Nooran’s Patch 1, while the fourth axis highlighted a strong differentiation of samples collected from Lake Noonamah’s Patch 1 and accounted for a further 7.8% of the total variation (Figure 3b). 

The structure analysis showed four likely genetic clusters in *M. drummondii* (Figure 3c). The first cluster consisted of all individuals from Lake Nooran’s Patch 1, seven samples from Lake Noonamah’s Patch 1, and two samples from Lake Noonamah’s Patch 2 (Figure 3d). The second cluster consisted of all samples from Lake Nooran’s Patch 2 and all samples from Oxley Lagoon’s Patch 2. The third cluster consisted of four samples from Lake Noonamah’s Patch 1, eleven (of thirteen) from Lake Noonamah’s Patch 2, and all (thirteen) samples from Lake Noonamah’s Patch 3. The fourth cluster consisted of all (13) samples from Oxley Lagoon’s Patch 1 and two samples from Oxley Lagoon’s Patch 2.

**Figure 4:**
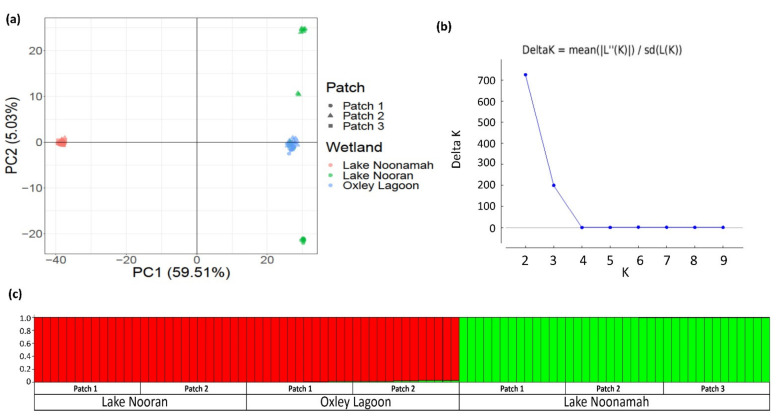
(**a**) Plot from PCoA analysis of 86 *E. acuta* genotypes across three wetlands (2–3 patches per wetland) in the lower Lachlan River Catchment; (**b**) representation of the Akaike value used to determine the number of populations (K); and (**c**) representation of the probability of population assignment with each individual genotype, coloured by cluster and ordered by wetland and patch.

The *E. acuta* PCoA axis 1 accounted for the majority of the variation (59.5%) and highlighted a strong differentiation between samples collected from Lake Noonamah and those from Oxley Lagoon and Lake Nooran (Figure 4a). The PCoA axis 2 accounted for 5% of the variation and highlighted the differentiation between samples collected from Lake Nooran’s Patch 1 and Patch 2. The third axis (not shown) accounted for 4.2% of the variation and differentiation between the samples collected from Oxley Lagoon and Lake Nooran. This PCoA also highlighted strong within-patch clustering of samples collected from almost all of the patches. The structure analysis confirmed these two genetic clusters (Figure 4b), with the largest group consisting of all samples collected from Oxley Lagoon and Lake Nooran and the other group consisting of all 39 samples collected from within Lake Noonamah (Figure 4c).

A hierarchical AMOVA demonstrated that the majority of the variation was attributed to that within individuals (64% for *M. drummondii* and 67% for *E. acuta*), which was significant in both species (Table 4). A total of 31% and 32% of the variation in *M. drummondii* and *E. acuta* was attributed to variation among wetlands, respectively, which was significant in both species (Table 4). The variation between samples within wetlands was estimated to be 5% in *M. drummondii* and 1% in *E. acuta*, which were both non-significant (Table 4).

The pairwise *F*_ST_ values for *M. drummondii* were 0.10 between Oxley Lagoon and Lake Nooran, 0.11 between Lake Nooran and Lake Noonamah, and 0.12 between Oxley Lagoon and Lake Noonamah. The pairwise *F*_ST_ values for *E. acuta* were just 0.03 between Oxley Lagoon and Lake Nooran, 0.23 between Oxley Lagoon and Lake Noonamah, and 0.25 between Lake Nooran and Lake Noonamah. The pairwise *F*_ST_ between patches was higher for both species than the comparisons at the wetland scale. For *M. drummondii*, the *F*_ST_ ranged from almost no variation (*F*_ST_ = 0.03) between Lake Noonamah’s Patch 2 and Lake Noonamah’s Patch 3 to the highest difference between Lake Nooran’s Patch 1 and Lake Noonamah’s Patch 3 (*F*_ST_ = 0.40). For *E. acuta*, the *F*_ST_ ranged from 0.03 between Lake Noonamah’s Patches 1 and 2 to ~0.31 between all the Lake Noonamah patches and Lake Nooran’s Patches 1 and 2 (Table 5).

## 4. Discussion

The results here demonstrate that, as with most aquatic plants [1], asexual vegetative reproduction occurs in *M. drummondii* and *E. acuta*, with samples of both species having identical genotypes. Unexpectedly, however, the relatively low occurrence of identical genotypes in both species suggests that this is not the major reproductive strategy of either species. Whilst *M. drummondii* and *E. acuta* grow clonally through rhizomes, the extent of clonality and the size and arrangement of genotypes varied between the two species.

Seven samples contributed to the same genotype in *M. drummondii*, which were collected in one patch within Lake Nooran, highlighting the potential extent (approximately 4 square metres) of a single genet (clone) in this species. A genotype in Lake Noonamah was sampled from two patches within the same wetland, highlighting the potential ability (albeit uncommon based on our data) of asexual dispersal via vegetative propagules within a wetland. In *E. acuta*, only one pair of identical samples was detected within a patch at a distance of 50 cm, demonstrating that vegetative reproduction also occurs in this species; however, the size of an individual genet is no more than 50 cm and is likely to be most commonly smaller.

Although the results show that asexual vegetative reproduction does occur, the dispersal of asexual propagules away from the parent plant is rare in *M. drummondii* and unlikely in *E. acuta*, and may be considered lower than what would be expected in aquatic macrophytes [2,17]. Boedeltje et al. [17] found that 90% of the hydrochorous propagules in streams in the Netherlands were vegetative. However, many species showed a preference for either vegetative or generative dispersal; for example, *Phragmites australis* was recorded predominately from generative (sexual) diaspores while *Potamogeton natans* reproduced exclusively by vegetative propagules [59]. The vegetative growth in *E. acuta* and *M. drummondii* is through the production of rhizomes, which have a low dispersibility compared to other vegetative organs such as stolons, runners, and bulbils [12]. However, rhizomes of some aquatic plants, such as *Phragmites australis*, can float for at least six months under experimental conditions once removed from the soil [60]. For vegetative dispersal to occur in *E. acuta* and *M. drummondii*, a combination of destructive removal (such as by birds) coinciding with flooding conditions may be required.

Sexual reproduction appears to be the dominant reproductive strategy in both species, and patches consisted of numerous genets. This suggests that patches are regularly invaded by additional genotypes of sexual origin and that genets are rarely large and dominant. Both *E. acuta* and *M. drummondii* have persistent soil seed or spore banks [40,43] and these results suggest a reliance on these soil banks rather than on vegetative reproduction for population persistence.

The reliance on sexual reproduction rather than vegetative shown here may be related to the infrequent and variable flooding regime and the extended dry phases experienced on river floodplain systems in dryland regions such as the lower Lachlan River [30], compared with aquatic plants in more permanent hydrological regimes. Persistent soil seed banks are known to be important for enabling aquatic plants to persevere on floodplains in dryland regions [61]. A similar situation is likely to exist in the water fern examined here, since *M. strigosa* growing in the Mediterranean basin was found to rely on sexual rather than vegetative reproduction, with the authors suggesting that sporocarps were the only means for this species to survive the harsh winter conditions [62]. The results from this study demonstrate the likely importance of sexual reproduction and the ability of sporocarps and seeds to remain viable during often-extended dry periods for the survival of *M. drummondii* and *E. acuta* on dryland river floodplains. The longevity of *E. acuta* seeds and *M. drummondii* sporocarps may help to reduce genetic drift and retain genetic variation in populations.

As with self-compatible flowering plants, heterosporous ferns such as *M. drummondii* produce both male and female sex organs, megaspores (male spores), and microspores (female spores), and as such, they are partially hermaphroditic and have the ability to self-fertilise [38]. As these are heterosporous, intragametophytic self-fertilisation (sperm and egg from one gametophyte producing the sporophyte) is not possible since the egg and sperm are on separate gametophytes. However, intergametophytic self-fertilisation, involving separate gametophytes derived from the one sporophyte, can occur. Our relatedness analyses provide strong evidence that *M. drummondii* can and does reproduce via self-fertilisation, with nine samples primarily from the same site in parent–offspring relationships via self-fertilisation. Self-fertilisation has been demonstrated in *M. strigosa* in the Mediterranean [62]. Interestingly, reproduction via self-fertilisation only occurred within Lake Noonamah, highlighting that differences between wetlands in landscape and hydrological processes may influence the rate of self-fertilisation in *M. drummondii* and potentially other species where reproduction occurs in water. Among the population variation in plant mating systems (self-fertilisation vs. exclusive outcrossing), within-species variation has been observed in other plants [63].

In *E. acuta,* only one pair of samples was in a parent–offspring relationship through self-fertilisation, suggesting that while this species may reproduce via self-fertilisation, mechanisms to reduce self-fertilisation such as dichogamy, as observed in *E. laeviglumis* [46], exist. Other mechanisms to promote outcrossing also exist in the *Cyperaceae*, such as self-incompatibility in *Rhynchospora ciliata* [64].

During a reproductive experiment of multiple *Marsilea* species, Schneider and Pryer [38] observed that submerged sporocarps sank and then opened within a few hours, followed by the simultaneous release of megaspores and microspores, which drifted to the water surface where fertilisation occurred over approximately 10 h. This suggests that *M. drummondii* has no apparent adaptation to reduce selfing, apart from the opportunistic mixing of spores. The greater the number of spores from different genotypes on the water surface, the more likely is cross-pollination.

The measures of within-wetland genetic diversity found in *M. drummondii* and *E. acuta* were both highest at Oxley Lagoon, intermediate at Lake Nooran, and lowest at Lake Noonamah. These similar patterns, despite different life histories, highlight how genetic diversity in the aquatic plants of dryland river floodplains are driven by numerous factors, including geographic barriers and the hydrological regime. Oxley Lagoon is situated in very close proximity to the Lachlan River (<50 m) and Lake Nooran is approx. 200 m away, while Lake Noonamah is >5 km from the main channel, suggesting that the distance from the river may have influenced the levels of genetic diversity. Genetic diversity was found to increase in downstream populations of *Sparganium emersum* (unbranched bur-reed), which was related to the effects of unidirectional downstream flow on diversity patterns [65]. Similarly, our results suggest that genetic diversity decreases the further from the main channel a population is located. 

The high levels of genetic differentiation and clustering within patches and wetlands suggests that pollination and dispersal most likely occur in proximity to the parent plants. Colonisation (founder) events between wetlands appear to be very rare in *E. acuta*, but more frequent in *M. drummondii*. This high genetic differentiation further suggests that colonisation (or recolonisation following localised extinction) occurs from the local propagule pool (deme/wetland), and that the mixing of colonising individuals from different demes is unlikely [16]. The limited gene flow from either sexual or asexual propagules in these semi-aquatic species is likely a key driver of population genetic structure by promoting genetic differentiation. The possible influence of local conditions in the fixing of alleles in different wetlands and even different patches [66], as well as founder effects, are also likely to influence the population genetic structure observed.

Fifty percent of *E. acuta* seeds floated for at least 30 days in an experiment by Higgisson and Dyer [43], indicating a capacity for long-distance dispersal under suitable conditions. Whilst potential dispersal may be much greater in *E. acuta*, here we show that the realised seed dispersal is most likely within close proximity to the parent plant and within a wetland. Although the hydrochorous dispersal of sporocarps is unknown, these start to open within a few hours of contact with water [38], possibly limiting long-distance hydrochory. While our results suggest that dispersal is most likely to occur within close proximity of the parent plant, the finding that some *M. drummondii* individuals were scattered beyond the patch they were sampled from in the PCoA suggests that gene flow is occurring between wetlands; this situation was not observed for *E. acuta*. 

The floodplain of the lower Lachlan River is known as a nesting and feeding habitat for a range of waterbirds, which have been recorded traveling along the Lachlan River and between its wetlands [67]. In the absence of specific information on *M. drummondii*, the sporocarps of *M. mucronata* have been shown to pass intact through the digestive tract of waterbirds [41], showing the likely role of waterbirds in their dispersal. Whilst intact seeds of *E. acuta* were recorded in faecal samples of the Grey Teal waterbird, none of these seeds germinated during experimental trials [44]. A lowered seed viability following ingestion by waterbirds may have contributed to the lower connectivity and gene flow between wetlands shown here in *E. acuta*, compared with *M. drummondii*.

## 5. Conclusions

While asexual vegetative reproduction does occur in *M. drummondii* and *E. acuta*, sexual reproduction is the dominant reproductive strategy. Dispersal between wetlands (and even between patches) is infrequent in *M. drummondii* and extremely rare in *E. acuta*. This suggests a reliance on local seed sources and seed or spore bank longevity. Low dispersal means that recolonising wetland patches is challenging, especially under a drying climate across the study region where the timing, frequency, and volume of precipitation is likely to dramatically change. In river floodplain systems in dryland regions, such as the lower Lachlan River, the recolonisation of patches following a disturbance such as an extended drought may be unlikely. These results highlight the importance of protecting existing populations and maintaining diverse and functioning wetlands. There may also be a need to collect and store propagules for future restoration activities. Once lost from a wetland, these species may not have the ability to recolonise, especially under regulated flow conditions.

## Figures and Tables

**Table 1:** Pairwise relatedness scenarios used as part of this study and the possible options at given loci.

(1) Genetically Identical Pairs through Asexual Reproduction		(2) Pairs in a Parent–Offspring Rel. through Self-Fertilisation		(3) Pairs in a Parent–Offspring Relationship through Outcrossing	
Possible Loci	Can Be:	Possible Loci	Can Be:	Possible Loci	Can Be:
AA	AA	AA	AA	AA	AB, AA
AB	AB	AB	AA, AB, BB	AB	AA, AB, BB
BB	BB	BB	BB	BB	AB, BB

**Table 2:** Number of genotypes within each wetland and patch for *Marsilea drummondii* (MD) and *Eleocharis acuta* (EA). The numbers in brackets are the numbers of samples collected.

		*(n)* Genotypes			
Wetland	Patch	MD		EA	
		Wetland	Patch	Wetland	Patch
Lake Nooran	1	21 (26)	7 (13)	26 (26)	13 (13)
	2		13 (13)		13 (13)
Noonamah Wetland	1	37 (39)	12 (13)	38 (39)	13 (13)
	2		13 (13)		12 (13)
	3		13 (13)		13 (13)
Oxley Lagoon	1	26 (26)	13 (13)	26 (26)	13 (13)
	2		13 (13)		13 (13)
All—Lachlan		83 (91)		90 (91)	

**Table 3:** Observed (*H_o_*) and expected (*H_e_*) genetic diversity within wetlands, patches, and overall for *Marsilea drummondii* (MD) and *Eleocharis acuta* (EA). F_IS_ = (median) inbreeding coefficient.

		*H_o_*				*H_e_*				F_IS_	
Wetland	Patch	MD		EA		MD		EA		MD	EA
		Wetland	Patch	Wetland	Patch	Wetland	Patch	Wetland	Patch		
Lake Nooran	1	0.117	0.035	0.244	0.248	0.105	0.021	0.190	0.172	−0.669	−0.403
	2		0.160		0.241		0.093		0.173	−0.693	−0.354
Lake Noonamah	1	0.035	0.045	0.060	0.056	0.050	0.059	0.056	0.051	0.264	−0.076
	2		0.040		0.063		0.050		0.056	0.229	−0.090
	3		0.019		0.061		0.013		0.051	−0.362	−0.157
Oxley Lagoon	1	0.205	0.278	0.239	0.244	0.203	0.189	0.193	0.188	−0.439	−0.265
	2		0.128		0.233		0.125		0.183	0.018	−0.236
All—Lachlan		0.105		0.165		0.144		0.220			

**Table 4:** Analysis of molecular variance (AMOVA) for *M. drummondii* and *E. acuta*. * = significant with *p* < 0.05.

Source of Variation	df	Sum of Squares	Mean Sum of Squares	Estimated (%) Variation	*p* Value
*M.* *drummondii*					
Between wetlands	2	7092	3546	31%	0.01 *
Between samples within wetlands	81	12,227	150	5%	0.12
Within samples	84	10,943	130	64%	0.01 *
Total	167	30,262	181	100%	
*E.* *acuta*					
Between wetlands	2	64,774	32,387	32%	0.01 *
Between samples within wetlands	87	99,304	1141	1%	0.34
Within samples	90	98,384	1093	67%	0.01 *
Total	179	262,462	1466	100%	

**Table 5:** F_ST_ values for each patch for *M. drummondii* on the bottom left and *E. acuta*, on the top right of the table.

	Lake Nooran 1	Lake Nooran 2	Noonamah 1	Noonamah 2	Noonamah 3	Oxley 1	Oxley 2
**Lake Nooran 1**	---	0.075	0.307	0.301	0.309	0.058	0.055
**Lake Nooran 2**	0.334	---	0.311	0.305	0.313	0.062	0.057
**Noonamah 1**	0.339	0.277	---	0.033	0.046	0.279	0.275
**Noonamah 2**	0.165	0.167	0.066	---	0.040	0.274	0.270
**Noonamah 3**	0.395	0.308	0.155	0.026	---	0.281	0.276
**Oxley 1**	0.318	0.266	0.277	0.201	0.303	---	0.039
**Oxley 2**	0.173	0.031	0.158	0.116	0.170	0.199	---

## Data Availability

The genetic and metadata have been provided to the journal as part of the submission of this manuscript and have also been archived in the University of Canberra’s Institute for Applied Ecology data repository, and are available upon request. The R code that we developed to determine the pairwise samples in each of the three relatedness scenarios is also available upon request.

## Supplementary Materials

The following supporting information can be downloaded at: https://www.mdpi.com/article/10.3390/genes13091506/s1, Figure S1: A graphical representation of the relatedness in *Marsilea drummondii*; Figure S2: A graphical representation of the relatedness in *Eleocharis acuta*. Table S1: Results from pairwise relatedness analyses for *M. drummondii*. Table S2: Results from pairwise relatedness analyses for *E. acuta*.
