# Supplementary material for: Reproductive Strategies and Population Genetic Structure in Two Dryland River Floodplain Plants, Marsilea drummondii and Eleocharis acuta
Source: Genes (Basel). 2022 Aug 23;13(9):1506. doi: 10.3390/genes13091506 (PMC9498545; doi:10.3390/genes13091506)
Supplement: Supplementary file 1 [file genes-13-01506-s001.zip › genes-1842001- updated supplementary.pdf]

Supplementary material

Results from pairwise relatedness analysis based on the three relatedness scenarios (clonality, parent-offspring through self-fertilisation, and Parent-offspring through out crossing) for *E. acuta* (Table S1) and *M. drummondii* (Table S2). An approximate spatial arrangement of samples within each patch and wetland and the relationship between each sample are represented as Figure S1 and Figure S2.

In each of the relatedness scenarios, based on 0 inconsistent loci, the green represents genetically identical pairs through vegetative reproduction, the red represents pairs in a parent-offspring relationship through self-fertilisation, and the yellow represents pairs in a parent offspring relationship through outcrossing. The thick black lines in Appendix b represent a potential cut-off based on the number of loci differences where pairs above the black lines may also be in the corresponding relatedness scenario, however, have some loci differences (<5) maybe related to genotyping errors. There was no obvious cut off in *M. drumondii* (Table S1). Only pairs with no (0) or few loci differences are shown here given the large number of pairs. The number of loci were the number of loci used in the pairwise analysis for those two samples (individuals).

Table S1. results from pairwise relatedness analysis *M. drummondii*.

Individual 1	Individual 2	number of loci	clonality	self-fertilisation	parent-offspring
Noonamah1-1	Noonamah1-12	1240	0	0	0
Noonamah1-1	Noonamah2-3	1240	0	0	0
Lake Nooran1-1	Lake Nooran1-2	1241	0	0	0
Lake Nooran1-1	Lake Nooran1-3	1241	0	0	0
Lake Nooran1-1	Lake Nooran1-4	1241	0	0	0
Lake Nooran1-1	Lake Nooran1-5	1240	0	0	0
Lake Nooran1-1	Lake Nooran1-6	1241	0	0	0
Lake Nooran1-1	Lake Nooran1-7	1241	0	0	0
Lake Nooran1-2	Lake Nooran1-3	1241	0	0	0
Lake Nooran1-2	Lake Nooran1-4	1241	0	0	0
Lake Nooran1-2	Lake Nooran1-5	1240	0	0	0
Lake Nooran1-2	Lake Nooran1-6	1241	0	0	0
Lake Nooran1-2	Lake Nooran1-7	1241	0	0	0
Noonamah1-12	Noonamah2-3	1241	0	0	0
Lake Nooran1-3	Lake Nooran1-4	1241	0	0	0
Lake Nooran1-3	Lake Nooran1-5	1240	0	0	0
Lake Nooran1-3	Lake Nooran1-6	1241	0	0	0
Lake Nooran1-3	Lake Nooran1-7	1241	0	0	0
Lake Nooran1-4	Lake Nooran1-5	1240	0	0	0
Lake Nooran1-4	Lake Nooran1-6	1241	0	0	0
Lake Nooran1-4	Lake Nooran1-7	1241	0	0	0
Lake Nooran1-5	Lake Nooran1-6	1240	0	0	0
Lake Nooran1-5	Lake Nooran1-7	1240	0	0	0
Lake Nooran1-6	Lake Nooran1-7	1241	0	0	0
Noonamah1-1	Noonamah1-6	1237	1	0	0
Noonamah1-10	Noonamah1-6	1234	1	0	0

Noonamah1-12	Noonamah1-6	1238	1	0	0
Noonamah2-2	Noonamah2-3	1239	1	0	0
Noonamah1-8	Noonamah2-3	1241	1	0	0
Noonamah3-11	Noonamah2-3	1241	5	0	0
Noonamah3-11	Noonamah1-6	1238	6	0	0
Noonamah2-5	Noonamah1-12	1236	8	0	0
Noonamah2-5	Noonamah2-3	1236	8	0	0
Noonamah2-5	Noonamah1-6	1233	9	0	0
Noonamah1-1	Noonamah2-2	1238	1	1	0
Noonamah1-1	Noonamah1-8	1240	1	1	0
Noonamah1-11	Noonamah1-4	1241	1	1	0
Noonamah1-11	Noonamah1-7	1241	1	1	0
Noonamah1-12	Noonamah2-2	1239	1	1	0
Noonamah1-12	Noonamah1-8	1241	1	1	0
Noonamah1-6	Noonamah2-3	1238	1	1	0
Noonamah1-1	Noonamah1-10	1234	2	1	0
Noonamah1-10	Noonamah1-12	1235	2	1	0
Noonamah1-10	Noonamah2-3	1235	2	1	0
Noonamah1-4	Noonamah1-7	1241	2	1	0
Noonamah2-2	Noonamah1-8	1239	2	1	0
Noonamah1-1	Noonamah2-1	1240	3	1	0
Noonamah1-3	Noonamah1-11	1241	3	1	0
Noonamah1-12	Noonamah2-1	1241	3	1	0
Noonamah1-11	Noonamah1-13	1240	5	1	0
Lake Nooran1-1	Lake Nooran1-11	1219	6	1	0
Lake Nooran1-2	Lake Nooran1-11	1219	6	1	0
Noonamah1-4	Noonamah1-13	1240	6	1	0
Noonamah3-11	Noonamah2-2	1239	6	1	0
Noonamah3-11	Noonamah1-8	1241	6	1	0
Noonamah3-1	Noonamah2-1	1238	7	1	0
Noonamah3-9	Noonamah1-6	1228	7	1	0
Noonamah2-9	Noonamah2-3	1232	7	1	0
Noonamah2-4	Noonamah1-6	1231	8	1	0
Noonamah2-6	Noonamah1-12	1236	8	1	0
Noonamah2-6	Noonamah2-3	1236	8	1	0
Noonamah3-11	Noonamah2-1	1241	8	1	0
Noonamah3-5	Noonamah2-3	1241	8	1	0
Noonamah2-5	Noonamah2-2	1234	9	1	0
Noonamah2-5	Noonamah1-8	1236	9	1	0
Noonamah2-6	Noonamah1-6	1233	9	1	0
Noonamah2-7	Noonamah2-3	1231	9	1	0
Noonamah2-7	Noonamah1-6	1228	10	1	0
Noonamah2-5	Noonamah2-1	1236	11	1	0
Noonamah1-6	Noonamah2-2	1236	2	2	0
Noonamah1-6	Noonamah1-8	1238	2	2	0
Noonamah1-10	Noonamah2-2	1233	3	2	0

Noonamah1-10	Noonamah1-8	1235	3	2	0
Noonamah2-1	Noonamah2-3	1241	3	2	0
Noonamah1-3	Noonamah1-4	1241	4	2	0
Noonamah1-3	Noonamah1-7	1241	4	2	0
Noonamah1-6	Noonamah3-13	1234	4	2	0
Noonamah1-1	Noonamah3-13	1236	5	2	0
Noonamah1-10	Noonamah3-13	1231	5	2	0
Noonamah1-12	Noonamah3-13	1237	5	2	0
Noonamah2-2	Noonamah3-13	1235	6	2	0
Noonamah3-3	Noonamah1-6	1208	7	2	0
Noonamah3-3	Noonamah2-3	1209	7	2	0
Noonamah2-10	Noonamah2-3	1223	7	2	0
Noonamah1-3	Noonamah1-13	1240	8	2	0
Noonamah3-1	Noonamah1-12	1238	8	2	0
Noonamah3-1	Noonamah2-3	1238	8	2	0
Noonamah3-9	Noonamah1-12	1231	8	2	0
Noonamah3-9	Noonamah2-3	1231	8	2	0
Noonamah2-9	Noonamah2-2	1230	8	2	0
Noonamah2-9	Noonamah1-8	1232	8	2	0
Noonamah2-4	Noonamah1-10	1228	9	2	0
Noonamah2-4	Noonamah1-12	1234	9	2	0
Noonamah2-4	Noonamah2-3	1234	9	2	0
Noonamah2-6	Noonamah2-2	1234	9	2	0
Noonamah2-6	Noonamah1-8	1236	9	2	0
Noonamah3-5	Noonamah1-8	1241	9	2	0
Noonamah2-7	Noonamah2-2	1229	10	2	0
Noonamah2-7	Noonamah1-8	1231	10	2	0
Noonamah3-10	Noonamah2-3	1235	10	2	0
Noonamah3-11	Noonamah3-13	1237	10	2	0
Noonamah2-6	Noonamah2-1	1236	11	2	0
Noonamah3-10	Noonamah1-6	1232	11	2	0
Noonamah2-7	Noonamah2-1	1231	12	2	0
Noonamah3-12	Noonamah2-3	1211	12	2	0
Noonamah2-5	Noonamah3-13	1232	13	2	0
Noonamah3-8	Noonamah1-12	1235	13	2	0
Noonamah3-8	Noonamah2-3	1235	13	2	0
Noonamah3-8	Noonamah1-6	1232	14	2	0
Noonamah3-8	Noonamah2-1	1235	14	2	0
Noonamah2-1	Noonamah2-2	1239	4	3	0
Noonamah2-1	Noonamah1-8	1241	4	3	0
Noonamah3-13	Noonamah2-3	1237	5	3	0
Noonamah3-3	Noonamah2-2	1207	8	3	0
Noonamah3-3	Noonamah1-8	1209	8	3	0
Noonamah2-10	Noonamah1-8	1223	8	3	0
Noonamah3-1	Noonamah2-2	1236	9	3	0
Noonamah3-1	Noonamah1-8	1238	9	3	0

Noonamah3-9	Noonamah2-2	1229	9	3	0
Noonamah3-9	Noonamah1-8	1231	9	3	0
Noonamah3-2	Noonamah2-3	1227	9	3	0
Lake Nooran1-12	Lake Nooran1-4	1230	9	3	0
Lake Nooran1-12	Lake Nooran1-5	1229	9	3	0
Lake Nooran1-12	Lake Nooran1-6	1230	9	3	0
Lake Nooran1-12	Lake Nooran1-7	1230	9	3	0
Noonamah3-3	Noonamah2-1	1209	9	3	0
Noonamah3-3	Noonamah3-13	1205	9	3	0
Noonamah2-4	Noonamah2-2	1232	10	3	0
Noonamah2-4	Noonamah1-8	1234	10	3	0
Noonamah3-2	Noonamah1-6	1224	10	3	0
Noonamah3-9	Noonamah3-13	1227	11	3	0
Noonamah3-10	Noonamah2-2	1233	11	3	0
Noonamah3-10	Noonamah1-8	1235	11	3	0
Noonamah2-4	Noonamah3-13	1230	12	3	0
Noonamah2-9	Noonamah3-13	1228	12	3	0
Noonamah2-6	Noonamah3-13	1232	13	3	0
Noonamah3-10	Noonamah2-1	1235	13	3	0
Noonamah3-12	Noonamah2-2	1209	13	3	0
Noonamah3-12	Noonamah1-8	1211	13	3	0
Noonamah3-5	Noonamah3-13	1237	13	3	0
Noonamah3-8	Noonamah2-2	1233	14	3	0
Noonamah3-8	Noonamah1-8	1235	14	3	0
Noonamah2-7	Noonamah3-13	1227	14	3	0
Noonamah3-12	Noonamah3-13	1207	16	3	0
Noonamah3-13	Noonamah1-8	1237	6	4	0
Lake Nooran1-1	Lake Nooran1-13	1222	10	4	0
Lake Nooran1-2	Lake Nooran1-13	1222	10	4	0
Noonamah3-2	Noonamah2-2	1225	10	4	0
Noonamah3-2	Noonamah1-8	1227	10	4	0
Lake Nooran1-3	Lake Nooran1-13	1222	10	4	0
Noonamah3-11	Noonamah2-10	1223	10	4	0
Lake Nooran1-4	Lake Nooran1-13	1222	10	4	0
Noonamah3-2	Noonamah2-1	1227	12	4	0
Noonamah3-2	Noonamah3-13	1223	12	4	0
Noonamah3-10	Noonamah3-13	1231	15	4	0
Noonamah3-8	Noonamah3-13	1231	18	4	0
Noonamah1-1	Noonamah3-11	1240	5	5	0
Noonamah1-12	Noonamah3-11	1241	5	5	0
Lake Nooran1-11	Lake Nooran1-3	1219	6	5	0
Lake Nooran1-11	Lake Nooran1-4	1219	6	5	0
Lake Nooran1-11	Lake Nooran1-5	1218	6	5	0
Lake Nooran1-11	Lake Nooran1-6	1219	6	5	0
Lake Nooran1-11	Lake Nooran1-7	1219	6	5	0
Noonamah1-13	Noonamah1-7	1240	6	5	0

Noonamah1-1	Noonamah3-3	1208	7	5	0
Noonamah1-1	Noonamah2-10	1222	7	5	0
Noonamah1-12	Noonamah3-3	1209	7	5	0
Noonamah1-12	Noonamah2-10	1223	7	5	0
Noonamah1-10	Noonamah3-3	1205	8	5	0
Noonamah2-2	Noonamah2-10	1221	8	5	0
Noonamah1-1	Noonamah2-8	1212	10	5	0
Noonamah1-12	Noonamah2-8	1213	10	5	0
Noonamah2-8	Noonamah1-8	1213	10	5	0
Noonamah2-8	Noonamah2-3	1213	10	5	0
Noonamah2-8	Noonamah2-1	1213	11	5	0
Lake Nooran1-1	Lake Nooran1-10	1209	12	5	0
Lake Nooran1-9	Lake Nooran1-1	1215	13	5	0
Lake Nooran1-9	Lake Nooran1-2	1215	13	5	0
Lake Nooran1-9	Lake Nooran1-3	1215	13	5	0
Lake Nooran1-9	Lake Nooran1-4	1215	13	5	0
Lake Nooran1-9	Lake Nooran1-5	1214	13	5	0
Lake Nooran1-9	Lake Nooran1-6	1215	13	5	0
Lake Nooran1-9	Lake Nooran1-7	1215	13	5	0
Noonamah2-5	Noonamah3-11	1236	13	5	0
Noonamah3-1	Noonamah2-8	1210	14	5	0
Noonamah3-7	Noonamah1-12	1211	15	5	0
Noonamah3-7	Noonamah2-3	1211	15	5	0
Noonamah2-5	Noonamah3-3	1204	15	5	0
Noonamah2-5	Noonamah2-10	1218	15	5	0
Noonamah3-7	Noonamah2-1	1211	16	5	0
Noonamah3-7	Noonamah1-5	1206	17	5	0
Noonamah2-5	Noonamah2-8	1208	18	5	0
Noonamah1-1	Noonamah2-9	1231	7	6	0
Noonamah1-10	Noonamah3-11	1235	7	6	0
Noonamah1-12	Noonamah2-9	1232	7	6	0
Noonamah1-1	Noonamah3-1	1237	8	6	0
Noonamah1-1	Noonamah3-9	1230	8	6	0
Noonamah1-10	Noonamah3-9	1225	8	6	0
Noonamah2-1	Noonamah2-10	1223	8	6	0
Noonamah1-1	Noonamah3-2	1226	9	6	0
Lake Nooran1-1	Lake Nooran1-12	1230	9	6	0
Lake Nooran1-2	Lake Nooran1-12	1230	9	6	0
Noonamah1-12	Noonamah3-2	1227	9	6	0
Lake Nooran1-3	Lake Nooran1-12	1230	9	6	0
Noonamah3-3	Noonamah2-10	1191	10	6	0
Lake Nooran1-13	Lake Nooran1-5	1221	10	6	0
Lake Nooran1-13	Lake Nooran1-6	1222	10	6	0
Lake Nooran1-13	Lake Nooran1-7	1222	10	6	0
Noonamah2-8	Noonamah2-2	1211	11	6	0
Noonamah3-11	Noonamah2-9	1232	12	6	0

Noonamah2-6	Noonamah3-11	1236	13	6	0
Noonamah3-1	Noonamah3-3	1207	13	6	0
Noonamah3-1	Noonamah2-10	1220	13	6	0
Noonamah3-2	Noonamah2-10	1209	13	6	0
Noonamah2-6	Noonamah2-10	1218	14	6	0
Noonamah3-9	Noonamah3-3	1199	14	6	0
Noonamah2-7	Noonamah3-11	1231	14	6	0
Noonamah2-9	Noonamah2-10	1214	14	6	0
Noonamah2-4	Noonamah3-3	1202	15	6	0
Noonamah2-5	Noonamah3-2	1222	15	6	0
Noonamah2-5	Noonamah2-9	1227	15	6	0
Lake Nooran1-10	Lake Nooran1-11	1189	15	6	0
Noonamah2-6	Noonamah3-3	1204	15	6	0
Noonamah2-7	Noonamah3-3	1199	15	6	0
Noonamah3-7	Noonamah2-2	1209	16	6	0
Noonamah3-7	Noonamah1-8	1211	16	6	0
Noonamah2-5	Noonamah3-1	1233	16	6	0
Noonamah2-5	Noonamah3-9	1226	16	6	0
Noonamah2-7	Noonamah2-10	1214	16	6	0
Noonamah3-8	Noonamah3-9	1226	17	6	0
Noonamah3-12	Noonamah2-10	1193	17	6	0
Noonamah2-6	Noonamah2-8	1208	18	6	0
Noonamah3-10	Noonamah2-8	1207	18	6	0
Lake Nooran1-9	Lake Nooran1-11	1196	19	6	0
Noonamah2-7	Noonamah2-8	1203	19	6	0
Noonamah3-8	Noonamah2-8	1208	21	6	0
Noonamah1-2	Noonamah1-11	1233	7	7	0
Noonamah1-1	Noonamah2-6	1235	8	7	0
Noonamah1-1	Noonamah3-5	1240	8	7	0
Noonamah1-12	Noonamah3-5	1241	8	7	0
Noonamah1-6	Noonamah2-9	1229	8	7	0
Noonamah1-1	Noonamah2-4	1233	9	7	0
Noonamah1-10	Noonamah2-9	1226	9	7	0
Noonamah2-2	Noonamah3-5	1239	9	7	0
Noonamah1-10	Noonamah3-2	1221	11	7	0
Lake Nooran1-10	Lake Nooran1-2	1209	12	7	0
Lake Nooran1-10	Lake Nooran1-3	1209	12	7	0
Lake Nooran1-10	Lake Nooran1-4	1209	12	7	0
Lake Nooran1-10	Lake Nooran1-5	1208	12	7	0
Lake Nooran1-10	Lake Nooran1-6	1209	12	7	0
Lake Nooran1-10	Lake Nooran1-7	1209	12	7	0
Noonamah3-3	Noonamah3-11	1209	12	7	0
Noonamah3-1	Noonamah3-11	1238	13	7	0
Noonamah3-9	Noonamah3-11	1231	13	7	0
Noonamah3-3	Noonamah2-9	1201	13	7	0
Noonamah3-11	Noonamah3-5	1241	13	7	0

Noonamah2-4	Noonamah3-11	1234	14	7	0
Noonamah3-2	Noonamah3-3	1195	14	7	0
Noonamah2-4	Noonamah3-9	1224	15	7	0
Noonamah2-6	Noonamah3-9	1226	15	7	0
Noonamah2-6	Noonamah2-9	1227	15	7	0
Noonamah3-10	Noonamah3-11	1235	15	7	0
Noonamah3-10	Noonamah2-9	1226	15	7	0
Noonamah2-5	Noonamah2-6	1231	16	7	0
Noonamah2-5	Noonamah3-10	1230	16	7	0
Noonamah2-5	Noonamah3-5	1236	16	7	0
Noonamah2-6	Noonamah3-1	1233	16	7	0
Noonamah2-7	Noonamah2-9	1222	16	7	0
Noonamah3-10	Noonamah3-3	1203	17	7	0
Noonamah3-10	Noonamah2-10	1217	17	7	0
Noonamah3-8	Noonamah3-11	1235	18	7	0
Noonamah2-7	Noonamah3-2	1217	18	7	0
Noonamah3-8	Noonamah3-1	1232	19	7	0
Lake Nooran1-12	Lake Nooran1-13	1212	19	7	0
Noonamah3-8	Noonamah3-3	1204	20	7	0
Noonamah3-8	Noonamah2-10	1217	20	7	0
Noonamah3-8	Noonamah3-2	1221	21	7	0
Noonamah1-1	Noonamah2-5	1235	8	8	0
Noonamah1-2	Noonamah1-4	1233	8	8	0
Noonamah1-2	Noonamah1-7	1233	8	8	0
Noonamah1-1	Noonamah2-7	1230	9	8	0
Noonamah1-12	Noonamah2-7	1231	9	8	0
Noonamah1-6	Noonamah3-5	1238	9	8	0
Noonamah1-1	Noonamah3-10	1234	10	8	0
Noonamah1-10	Noonamah2-6	1230	10	8	0
Noonamah1-10	Noonamah2-7	1225	10	8	0
Noonamah1-10	Noonamah3-5	1235	10	8	0
Noonamah1-12	Noonamah3-10	1235	10	8	0
Noonamah2-1	Noonamah2-9	1232	10	8	0
Noonamah1-2	Noonamah1-13	1233	12	8	0
Noonamah2-8	Noonamah3-3	1183	13	8	0
Noonamah3-2	Noonamah3-11	1227	14	8	0
Noonamah3-2	Noonamah2-9	1218	14	8	0
Noonamah2-10	Noonamah3-5	1223	14	8	0
Noonamah3-1	Noonamah2-9	1229	15	8	0
Noonamah3-9	Noonamah2-9	1222	15	8	0
Noonamah2-9	Noonamah3-5	1232	15	8	0
Noonamah2-4	Noonamah2-9	1225	16	8	0
Noonamah2-6	Noonamah3-5	1236	16	8	0
Noonamah3-10	Noonamah3-5	1235	16	8	0
Noonamah2-5	Noonamah2-7	1227	17	8	0
Noonamah3-1	Noonamah3-2	1224	17	8	0

Noonamah3-9	Noonamah3-2	1217	17	8	0
Noonamah2-7	Noonamah3-5	1231	17	8	0
Noonamah2-4	Noonamah3-2	1220	18	8	0
Noonamah3-2	Noonamah2-8	1199	18	8	0
Noonamah3-8	Noonamah2-6	1230	19	8	0
Noonamah3-8	Noonamah2-9	1226	20	8	0
Noonamah3-7	Noonamah2-8	1184	21	8	0
Lake Nooran1-9	Lake Nooran1-10	1187	21	8	0
Lake Nooran1-9	Lake Nooran1-13	1199	21	8	0
Lake Nooran2-3	Lake Nooran2-5	1239	53	8	0
Lake Nooran2-1	Lake Nooran2-9	1232	57	8	0
Noonamah1-2	Noonamah1-3	1233	10	9	0
Noonamah1-10	Noonamah2-5	1230	10	9	0
Noonamah1-1	Noonamah2-11	1240	11	9	0
Noonamah1-12	Noonamah2-11	1241	11	9	0
Noonamah2-1	Noonamah3-5	1241	11	9	0
Noonamah2-3	Noonamah2-11	1241	11	9	0
Noonamah1-10	Noonamah3-10	1229	12	9	0
Noonamah2-2	Noonamah2-11	1239	12	9	0
Noonamah1-8	Noonamah2-11	1241	12	9	0
Noonamah2-8	Noonamah2-10	1195	15	9	0
Noonamah3-3	Noonamah3-5	1209	15	9	0
Noonamah3-11	Noonamah3-12	1211	15	9	0
Noonamah3-1	Noonamah3-5	1238	16	9	0
Noonamah3-9	Noonamah3-5	1231	16	9	0
Lake Nooran1-11	Lake Nooran1-13	1201	16	9	0
Noonamah3-11	Noonamah2-11	1241	16	9	0
Noonamah2-9	Noonamah3-12	1202	16	9	0
Noonamah2-4	Noonamah2-6	1230	17	9	0
Noonamah2-4	Noonamah3-5	1234	17	9	0
Noonamah2-6	Noonamah2-7	1226	17	9	0
Noonamah2-9	Noonamah2-11	1232	17	9	0
Noonamah2-6	Noonamah3-10	1230	18	9	0
Noonamah2-5	Noonamah2-11	1236	19	9	0
Noonamah2-7	Noonamah3-10	1225	19	9	0
Noonamah3-7	Noonamah2-10	1193	20	9	0
Noonamah3-12	Noonamah3-5	1211	20	9	0
Noonamah3-7	Noonamah3-3	1181	21	9	0
Noonamah3-8	Noonamah3-5	1235	21	9	0
Noonamah1-1	Noonamah3-12	1210	12	10	0
Noonamah1-12	Noonamah3-12	1211	12	10	0
Noonamah1-6	Noonamah2-11	1238	12	10	0
Noonamah1-10	Noonamah2-11	1235	13	10	0
Noonamah1-1	Noonamah3-7	1210	15	10	0
Noonamah2-8	Noonamah3-11	1213	15	10	0
Noonamah2-10	Noonamah2-11	1223	16	10	0

Noonamah2-4	Noonamah2-5	1229	17	10	0
Noonamah3-1	Noonamah2-7	1228	17	10	0
Noonamah3-9	Noonamah2-7	1221	17	10	0
Noonamah3-2	Noonamah3-5	1227	17	10	0
Noonamah3-3	Noonamah2-11	1209	17	10	0
Noonamah2-4	Noonamah2-7	1224	18	10	0
Noonamah3-1	Noonamah3-10	1232	18	10	0
Noonamah3-9	Noonamah3-10	1225	18	10	0
Noonamah3-2	Noonamah3-10	1221	18	10	0
Noonamah2-4	Noonamah3-10	1228	19	10	0
Noonamah2-6	Noonamah2-11	1236	19	10	0
Noonamah2-7	Noonamah3-12	1201	19	10	0
Noonamah3-5	Noonamah2-11	1241	19	10	0
Noonamah3-7	Noonamah2-6	1206	20	10	0
Noonamah3-7	Noonamah3-11	1211	20	10	0
Noonamah2-5	Noonamah3-12	1206	20	10	0
Noonamah2-7	Noonamah2-11	1231	20	10	0
Noonamah3-10	Noonamah2-11	1235	20	10	0
Noonamah3-7	Noonamah3-1	1209	21	10	0
Noonamah3-12	Noonamah2-11	1211	21	10	0
Noonamah3-8	Noonamah2-7	1225	22	10	0
Noonamah3-8	Noonamah2-11	1235	22	10	0
Noonamah3-8	Noonamah3-10	1229	23	10	0
Lake Nooran2-3	Lake Nooran2-7	1238	38	10	0
Lake Nooran2-12	Lake Nooran2-10	1238	45	10	0
Lake Nooran2-3	Lake Nooran2-11	1234	50	10	0
Noonamah1-1	Noonamah3-8	1234	13	11	0
Noonamah1-6	Noonamah3-12	1208	13	11	0
Noonamah1-10	Noonamah3-12	1205	14	11	0
Noonamah2-1	Noonamah2-11	1241	14	11	0
Lake Nooran1-11	Lake Nooran1-12	1209	15	11	0
Noonamah2-8	Noonamah2-9	1204	17	11	0
Noonamah3-1	Noonamah2-11	1238	19	11	0
Noonamah3-9	Noonamah2-11	1231	19	11	0
Noonamah2-4	Noonamah2-11	1234	20	11	0
Noonamah2-6	Noonamah3-12	1208	20	11	0
Noonamah3-10	Noonamah3-12	1205	20	11	0
Noonamah2-5	Noonamah3-8	1230	21	11	0
Noonamah3-7	Noonamah2-9	1204	22	11	0
Lake Nooran1-9	Lake Nooran1-12	1206	22	11	0
Noonamah3-8	Noonamah3-12	1205	23	11	0
Lake Nooran2-3	Lake Nooran2-2	1230	47	11	0
Lake Nooran2-1	Lake Nooran2-2	1230	50	11	0
Noonamah1-10	Noonamah3-8	1229	15	12	0
Noonamah2-1	Noonamah3-12	1211	15	12	0
Noonamah3-13	Noonamah2-11	1237	16	12	0

Noonamah2-8	Noonamah2-11	1213	17	12	0
Noonamah3-1	Noonamah3-12	1208	18	12	0
Noonamah2-8	Noonamah3-5	1213	18	12	0
Noonamah3-3	Noonamah3-12	1179	19	12	0
Noonamah2-4	Noonamah3-8	1228	20	12	0
Noonamah2-4	Noonamah3-12	1205	20	12	0
Noonamah3-9	Noonamah3-12	1201	20	12	0
Noonamah3-2	Noonamah2-11	1227	20	12	0
Noonamah3-7	Noonamah3-10	1205	23	12	0
Noonamah3-7	Noonamah3-5	1211	23	12	0
Lake Nooran2-3	Oxley2-13	1241	50	12	0
Lake Nooran2-3	Lake Nooran2-9	1232	62	12	0
Lake Nooran1-10	Lake Nooran1-12	1198	20	13	0
Noonamah3-2	Noonamah3-12	1197	21	13	0
Lake Nooran2-3	Lake Nooran2-1	1241	23	13	0
Noonamah3-7	Noonamah2-5	1206	23	13	0
Lake Nooran2-3	Lake Nooran2-12	1240	27	13	0
Lake Nooran2-12	Lake Nooran2-2	1229	50	13	0
Noonamah2-8	Noonamah3-12	1183	20	14	0
Noonamah3-7	Noonamah2-11	1211	26	14	0
Lake Nooran2-12	Lake Nooran2-7	1237	45	14	0
Lake Nooran2-3	Lake Nooran2-10	1239	53	14	0
Lake Nooran2-1	Oxley2-12	1229	86	14	0
Noonamah3-7	Noonamah3-6	1145	26	15	0
Noonamah3-7	Noonamah3-12	1183	27	15	0
Lake Nooran2-12	Lake Nooran2-9	1231	67	15	0
Lake Nooran2-12	Oxley2-2	1231	72	15	0
Lake Nooran2-3	Oxley2-2	1231	73	15	0
Lake Nooran2-3	Oxley2-12	1229	85	15	0
Lake Nooran2-3	Lake Nooran2-6	1212	89	15	0
Noonamah3-7	Noonamah3-8	1206	28	16	0
Lake Nooran2-12	Lake Nooran2-1	1240	28	16	0
Lake Nooran2-3	Oxley2-8	1238	49	16	0
Lake Nooran2-3	Lake Nooran2-13	1240	37	17	0
Lake Nooran2-13	Lake Nooran2-10	1238	58	18	0
Lake Nooran2-12	Lake Nooran2-13	1239	40	19	0
Lake Nooran2-13	Lake Nooran2-7	1237	53	19	0
Lake Nooran2-3	Lake Nooran2-8	1239	51	20	0
Lake Nooran2-13	Lake Nooran2-1	1240	36	21	0
Lake Nooran2-13	Lake Nooran2-8	1238	50	21	0
Lake Nooran2-13	Lake Nooran2-2	1229	64	21	0
Lake Nooran2-12	Lake Nooran2-8	1238	56	23	0
Oxley2-8	Oxley2-2	1228	80	27	0
Lake Nooran2-8	Lake Nooran2-2	1228	71	29	0
Lake Nooran2-7	Lake Nooran2-1	1238	39	30	0
Oxley2-8	Lake Nooran2-7	1235	61	30	0

Oxley2-13	Lake Nooran2-2	1230	61	31	0
Oxley2-13	Lake Nooran2-13	1240	47	35	0
Lake Nooran2-8	Lake Nooran2-1	1239	56	35	0
Oxley2-8	Lake Nooran2-1	1238	52	36	0
Oxley2-13	Lake Nooran2-12	1240	51	38	0
Oxley2-13	Lake Nooran2-1	1241	49	39	0
Lake Nooran2-11	Lake Nooran2-12	1233	59	44	0
Lake Nooran2-5	Lake Nooran2-1	1239	52	46	0
Lake Nooran2-11	Lake Nooran2-13	1233	65	46	0
Lake Nooran2-4	Lake Nooran2-13	1237	67	49	0
Oxley1-6	Oxley1-1	1235	122	51	0
Oxley1-6	Oxley1-8	1238	81	57	0
Oxley2-7	Lake Nooran2-1	1221	73	63	0
Lake Nooran2-6	Lake Nooran2-2	1202	98	66	0
Oxley1-11	Oxley1-8	1237	94	74	0
Oxley1-7	Oxley1-8	1229	116	100	0

Table S2. results from pairwise relatedness analysis for *E. acuta*.

Individual1	Individual2	number of loci	clonality	self-fertilisation	parent-offspring
Noonamah2-1	Noonamah2-2	6593	0	0	0
Noonamah3-8	Noonamah3-12	6588	1	0	0
Noonamah1-2	Noonamah1-4	6604	2	2	0
Noonamah1-4	Noonamah1-5	6606	5	2	0
Noonamah1-2	Noonamah1-5	6604	5	3	0
Noonamah3-9	Noonamah3-10	6594	41	13	0
Noonamah3-10	Noonamah3-5	6602	24	23	0
Noonamah3-3	Noonamah3-4	6603	55	29	0
Noonamah3-1	Noonamah3-3	6605	60	29	0
Noonamah3-9	Noonamah3-5	6591	63	35	0
Noonamah1-1	Noonamah1-2	6548	63	36	0
Noonamah1-1	Noonamah1-4	6550	63	37	0
Noonamah1-1	Noonamah1-5	6550	64	37	0
Lake Nooran1-4	Lake Nooran1-6	6601	149	68	0
Lake Nooran1-3	Lake Nooran1-4	6607	148	71	0
Lake Nooran2-6	Lake Nooran2-2	6518	183	73	0
Oxley1-11	Oxley1-5	6545	139	74	0
Lake Nooran1-1	Lake Nooran1-4	6606	152	75	0
Lake Nooran1-11	Lake Nooran1-4	6605	150	76	0
Lake Nooran1-1	Lake Nooran1-11	6607	158	77	0
Lake Nooran1-11	Lake Nooran1-6	6601	165	77	0
Lake Nooran1-2	Lake Nooran1-6	6598	158	78	0

Lake Nooran1-2	Lake Nooran1-3	6605	147	82	0
Lake Nooran1-2	Lake Nooran1-4	6604	153	82	0
Lake Nooran1-2	Lake Nooran1-11	6603	159	84	0
Lake Nooran1-11	Lake Nooran1-3	6609	166	87	0
Oxley2-3	Oxley2-4	6556	191	90	0
Lake Nooran2-4	Lake Nooran2-5	6592	213	95	0
Lake Nooran2-4	Lake Nooran2-12	6583	208	102	0
Lake Nooran2-4	Lake Nooran2-9	6587	188	105	0
Lake Nooran2-12	Lake Nooran2-5	6589	231	106	0
Lake Nooran2-4	Lake Nooran2-1	6588	209	112	0
Lake Nooran2-12	Lake Nooran2-9	6581	219	122	0
Oxley1-4	Oxley1-1	6549	172	123	0
Lake Nooran2-3	Lake Nooran2-9	6589	228	134	0
Lake Nooran1-8	Lake Nooran1-4	6564	276	210	0
Noonamah3-1	Noonamah3-4	6603	64	33	1
Lake Nooran1-3	Lake Nooran1-6	6603	152	67	1
Oxley1-2	Oxley1-1	6561	120	69	1
Lake Nooran1-1	Lake Nooran1-6	6602	154	70	1
Lake Nooran1-1	Lake Nooran1-2	6605	160	74	1
Lake Nooran1-4	Lake Nooran1-7	6602	139	75	1
Lake Nooran1-3	Lake Nooran1-7	6605	149	77	1
Lake Nooran1-1	Lake Nooran1-3	6609	151	78	1
Lake Nooran2-8	Lake Nooran2-9	6591	174	80	1
Lake Nooran1-1	Lake Nooran1-7	6605	157	83	1
Lake Nooran1-11	Lake Nooran1-7	6603	157	85	1
Lake Nooran1-2	Lake Nooran1-7	6601	150	86	1
Lake Nooran1-6	Lake Nooran1-7	6599	150	87	1
Oxley2-7	Oxley2-2	6530	194	88	1
Lake Nooran1-9	Lake Nooran1-5	6522	194	90	1
Oxley1-8	Oxley1-9	6558	156	92	1
Oxley2-1	Oxley2-4	6559	179	92	1
Oxley2-7	Oxley2-11	6538	192	98	1
Oxley2-1	Oxley2-3	6554	182	99	1
Lake Nooran2-1	Lake Nooran2-9	6585	199	104	1
Oxley2-2	Oxley2-11	6530	201	112	1
Lake Nooran2-3	Lake Nooran2-4	6591	211	114	1
Oxley1-12	Oxley1-9	6543	183	115	1
Lake Nooran2-3	Lake Nooran2-5	6595	245	121	1
Lake Nooran2-5	Lake Nooran2-9	6589	208	127	1
Lake Nooran2-5	Lake Nooran2-1	6591	230	134	1
Lake Nooran1-8	Lake Nooran1-2	6562	277	205	1
Lake Nooran1-8	Lake Nooran1-3	6567	291	221	1
Lake Nooran2-11	Lake Nooran2-7	6520	174	85	2
Lake Nooran2-4	Lake Nooran2-8	6594	166	103	2
Oxley1-12	Oxley1-8	6538	195	108	2
Lake Nooran2-12	Lake Nooran2-8	6591	208	125	2

Lake Nooran2-3	Lake Nooran2-12	6588	239	127	2
Lake Nooran2-3	Lake Nooran2-8	6597	213	134	2
Lake Nooran2-3	Lake Nooran2-1	6591	240	137	2
Lake Nooran1-8	Lake Nooran1-7	6563	269	212	2
Oxley1-2	Oxley1-4	6538	218	83	3
Lake Nooran2-8	Lake Nooran2-1	6594	194	88	3
Lake Nooran2-12	Lake Nooran2-1	6585	225	123	3
Lake Nooran2-5	Lake Nooran2-8	6598	201	132	3
Lake Nooran1-8	Lake Nooran1-11	6565	270	207	3
Lake Nooran1-8	Lake Nooran1-6	6562	280	207	3
Lake Nooran1-8	Lake Nooran1-1	6566	269	209	3
Noonamah3-13	Noonamah3-6	6549	385	221	26
Noonamah2-4	Noonamah2-5	6565	445	207	29
Noonamah3-2	Noonamah3-10	6575	406	194	30

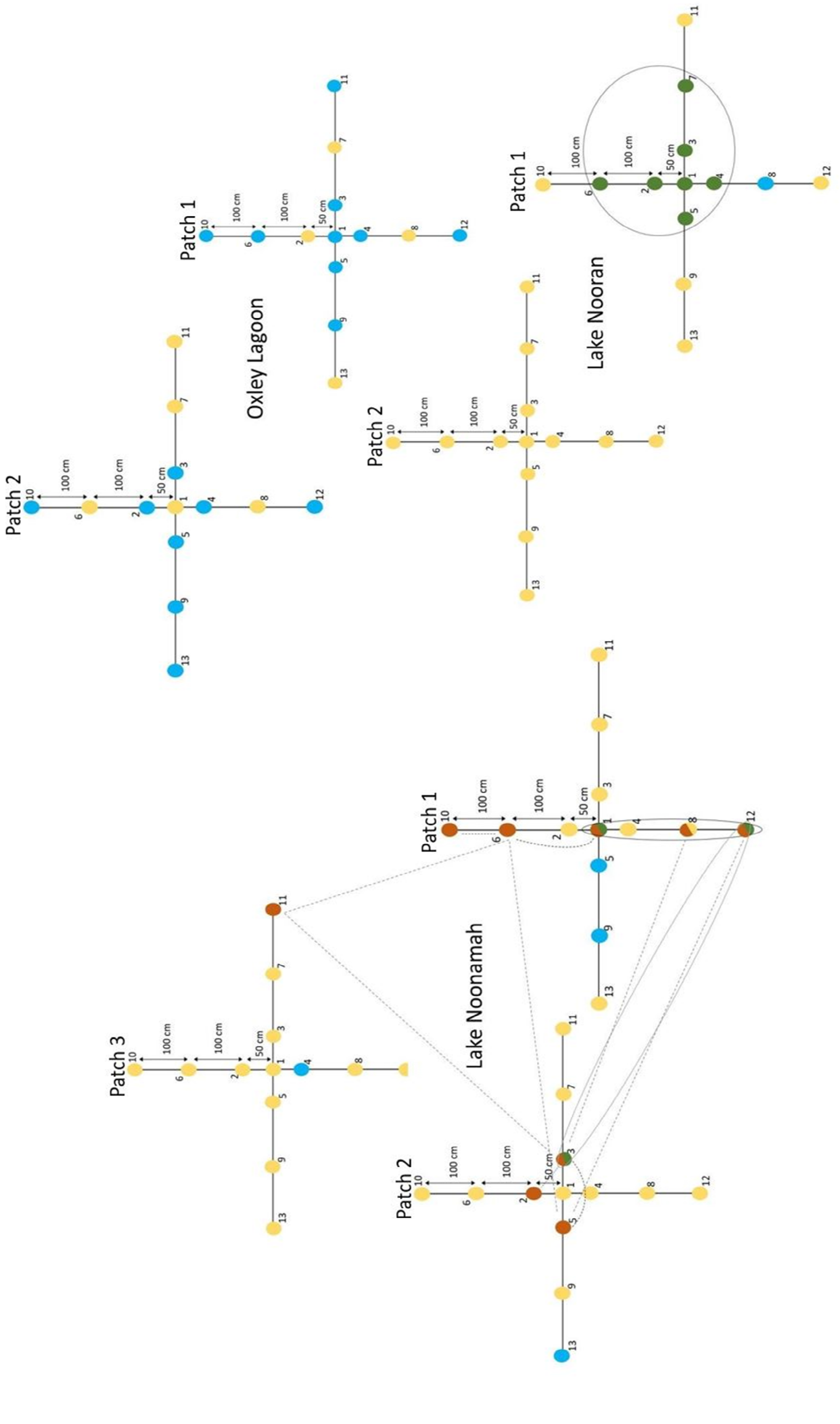

Figure S1. A graphical representation of the relatedness in *Marsilea drummondii.* The green circles represent identical genotypes through asexual vegetative reproduction and the grey circles show the size of each clone. The red circles represent a parent-offspring relationship through self-fertilisation and the dotted lines show the pairs in this relationship. The Yellow circles represents a parent off-spring relationship, and blue circles are not in a parent offspring relationship with any other of the samples.

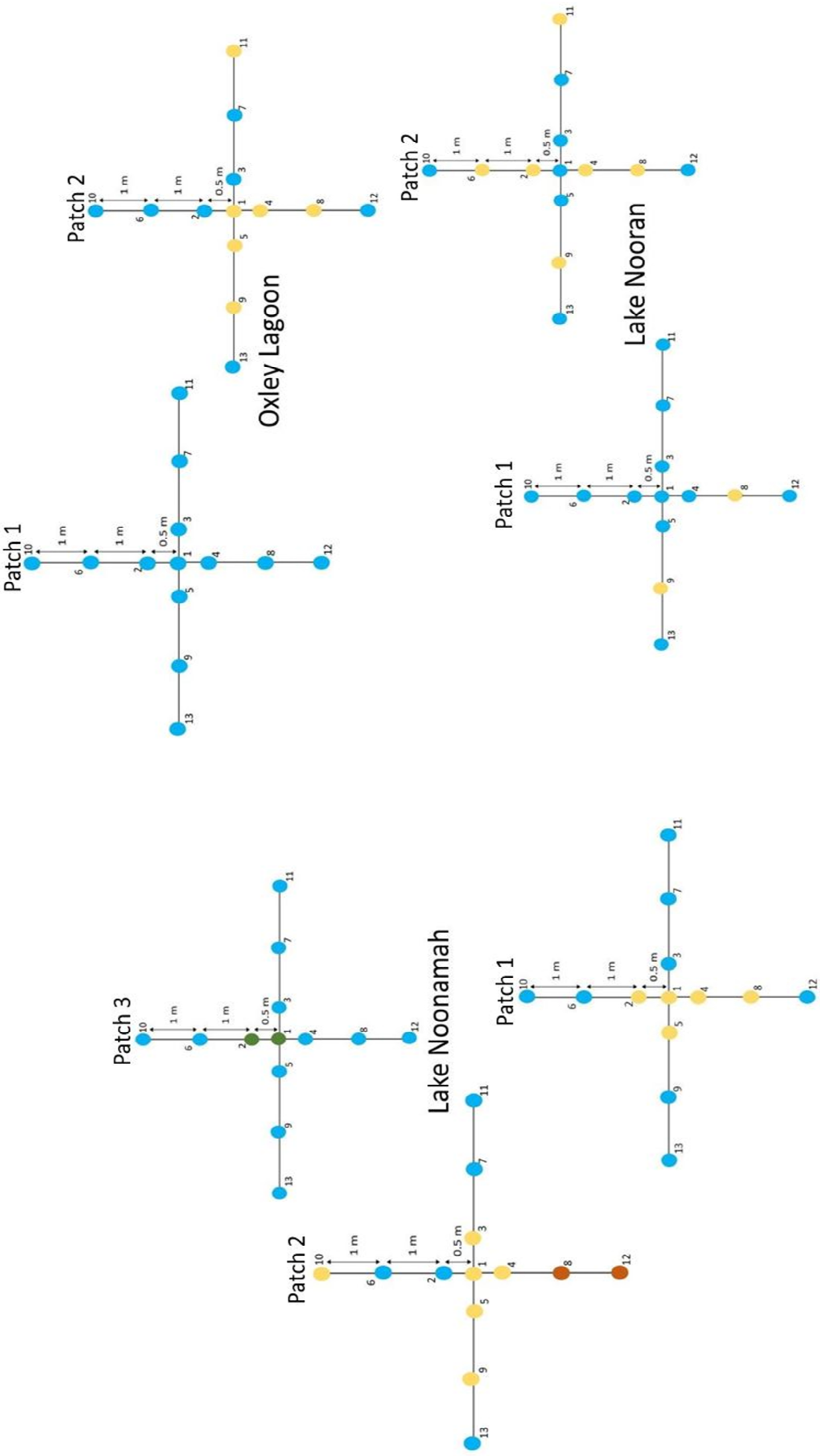

Figure S2. A graphical representation of the relatedness in *Eleocharis acuta.* The green circles represent identical genotypes through asexual vegetative reproduction. The red circles represent a parent-offspring relationship through self-fertilisation. The Yellow circles represents a parent off-spring relationship, and blue circles are not in a parent offspring relationship with any other of the samples. All pairs in these relationships occurred within and not between patches.
